# Characterization of pharmacogenetic markers related to Acute Lymphoblastic Leukemia toxicity in Amazonian native Americans population

**DOI:** 10.1038/s41598-020-67312-y

**Published:** 2020-06-24

**Authors:** Darlen Cardoso de Carvalho, Alayde Vieira Wanderley, André Mauricio Ribeiro dos Santos, Fabiano Cordeiro Moreira, Roberta Borges Andrade de Sá, Marianne Rodrigues Fernandes, Antonio André Conde Modesto, Tatiane Piedade de Souza, Amanda Cohen-Paes, Luciana Pereira Colares Leitão, Juliana Carla Gomes Rodrigues, Artur Luiz da Costa da Silva, João Farias Guerreiro, Sidney Santos, André Salim Khayat, Paulo Pimentel de Assumpção, Ney Pereira Carneiro dos Santos

**Affiliations:** 1 Oncology Research Nucleus, Universidade Federal Do Pará, Belém, PA Brazil; 2 Human and Medical Genetics Laboratory, Instituto de Ciências Biológicas, Universidade Federal Do Pará, Belém, PA Brazil; 3 Departamento de Pediatria, Ophir Loyola Hospital, Belém, PA Brazil; 4 Genomics and Bioinformatics Laboratory, Instituto de Ciências Biológicas, Universidade Federal Do Pará, Belém, Brazil; 5 João de Barros Barreto University Hospital, Universidade Federal Do Pará, Belém, PA Brazil; 6 Hospital Universitário João de Barros Barreto - Núcleo de Pesquisa Em Oncologia, 2º Piso da Unidade de Alta Complexidade Em Oncologia. Av. Mundurucus, 4487, Guamá, Belém, PA 66073-005 Brazil

**Keywords:** Cancer, Genetics, Molecular biology

## Abstract

Acute Lymphoblastic Leukemia (ALL) is the most common cancer in children. Differences are found among ethnic groups in the results of the treatment of pediatric ALL. In general, children with a high level of native American ancestry tend to respond less positively to ALL treatments, which may be related to specific genomic variants found in native American groups. Despite the evidence, few data are available on the distribution of the pharmacogenomic variants relevant to the treatment of ALL in traditional Amerindian populations, such the those of the Amazon region. Given this, the present study investigated 27 molecular markers related to the treatment of ALL in Amerindians from Brazilian Amazonia and compared the frequencies with those recorded previously on five continents, that are available in the 1,000 Genomes database. The variation in the genotype frequencies among populations was evaluated using Fisher’s exact test. The False Discovery Rate method was used to correct the results of the multiple analyses. Significant differences were found in the frequencies of the majority of markers between the Amerindian populations and those of other regions around the world. These findings highlight the unique genetic profile of the indigenous population of Brazilian Amazonia, which may reflect a distinct therapeutic profile for the treatment of ALL in these populations.

## Introduction

Acute Lymphoblastic Leukemia (ALL) is the most common cancer subtype found in children, accounting for almost 80% of cases^1,2^. The treatment of ALL is based on the application of a combination of chemotherapeutic agents. Two drugs, 6-mercaptopurine (6-MP) and Methotrexate (MTX), are the principal types of medication used during the consolidation and maintenance phases of the treatment of ALL. Survival rates are relatively high, at approximately 80%, although around 20% of the children treated present serious toxicological complications that frequently lead to the interruption of the treatment. The variation in the toxicological response of patients to the treatment of ALL may be determined by different polymorphic variants of the genes involved in the pharmacokinetics and/or pharmacodynamics of these drugs^3,4^. Ethnic differences have been recognized in many clinical studies of the treatment of ALL^5–10^. In general, Hispanic children present worse results in the treatment of ALL than European children^5–7,9^. This difference may be related to an increase in the frequency of variants of the germinative lineage associated with native American ancestry in Hispanic patients, which have a negative influence on the treatment of ALL^9^.

Despite the apparent influence of genetic variants linked to native American ancestry on the treatment of ALL in children, few data are available on the frequency of these variants in traditional Amerindian populations, such as those from the Amazon region^11^. The vast majority of the available studies have focused on European or American populations, which emphasizes the need to identify the genetic profile of Amerindian populations, in particular the variants that are significantly more common in these populations, as well as compiling a database for the development of clinical applications appropriate for this ethnic group and for populations admixed with this group, such as the general Brazilian population^11^.

In this context, the present study investigated 27 molecular markers related to the treatment of ALL in a combined Amerindian population from Brazilian Amazonia. These findings were compared with the data available on representative populations from five continents, that are available in the 1,000 Genomes database.

## Material and methods

### Study populations

The present study was approved by the Brazilian National Committee for Ethics in Research (CONEP), through protocol 961,451. The informed consent was obtained from each study participant and all research methods in this study were performed in accordance with approved guidelines and regulations. The study sample consisted of 203 healthy individuals from three Amerindian groups resident in the Brazilian Amazon region: 25 individuals from the Asurini do Koatinemo group, 84 individuals from the Asurini do Trocará group, and 94 from the Kayapó-Xicrin group. These individuals were combined in a single sample, denominated NAT (or Native), for the statistical analyses. For the comparisons with populations from other continents, data were obtained from the database of the 1,000 Genomes project, available at https://www.1000genomes.org. This sample (Supplementary Figure 1) included 661 individuals from Africa (AFR), 503 from Europe (EUR), 347 from the Americas (AMR), 504 from East Asia (EAS), and 489 from South Asia (SAS).

### Patients

Markers that showed a significantly different allelic distribution between Amerindian and of 1,000 Genomes populations were investigated in a sample of 42 patients diagnosed with B-cell ALL, from the Brazilian Amazon region and who had a high contribution of Amerindian genetic ancestry (see Supplementary Figure 2). For statistical analysis, these individuals were grouped as ALL_NAT. Diagnosis was confirmed based on the criteria of the French-American-British (FAB) classification system between the years of 2006 and 2018 in two reference public hospitals in the treatment of childhood cancer (Hospital Ophir Loyola and Hospital Oncológico Infantil Octavio Lobo, Belém-PA, Brazil), and peripheral blood samples were collected at the time of diagnosis. Clinical and demographic data of these patients are described in Supplementary Table 1.

### Selection of the genes and polymorphisms

A total of 27 genetic markers (Supplementary Table 2) were selected for the present study. All these markers (from the genes *ABCC1*, *ABCC2*, *ABCC3*, *AMPD1*, *ATIC*, *CCND1*, *GGH*, *ITPA*, *MTHFD1*, *MTHFR*, *MTRR*, *NALCN*, *NOS3*, *SHMT1*, *SLCO1B1*, *TLR4*, *TNFAIP3*, and *TPMT*) participate in the pharmacokinetics and/or pharmacodynamics of one or both of the drugs (6-MP or MTX) used to treat ALL. The polymorphisms were selected based on the information available in the PharmGKB, NCBI, and Ensembl databases, and through a literature search. The polymorphisms selected for the present study have potentially functional effects, including alterations of amino acids and the alternative splicing of promoter regions or are present in regions linked to transcription factors, as well as markers included in previous studies of ALL or the drugs used to treat this cancer.

### Genotyping of the polymorphisms and quality control

The genetic material was extracted from samples of peripheral blood, collected from the participants, using the commercial Biopur Mini Spin Plus–250 extraction kit (Biopur, Brazil), following the manufacturer’s instructions, and quantified using a NanoDrop 1,000 spectrophotometer (Termo Scientific NanoDrop 1,000; NanoDrop Technologies,Wilmington, DE). The polymorphisms were genotyped using the TaqMan OpenArray Genotyping technology (Applied Biosystems, Life Technologies, Carlsbad, CA) in a QuantStudio 12 K Flex Real-Time PCR System (Applied Biosystems, Life Technologies, Carlsbad, CA), following the protocol published by Applied Biosystems. The Taqman Genotyper software was used to analyze the data from the plates and the precision of the genotype reads, as well as to control the quality of the genotyping.

As fixed mutations are uninformative, the polymorphisms with Minor Allele Frequencies (MAFs) of less than 1% were omitted from the analyses, as were polymorphisms with no genotyping and absent genotyping rates of 20% or more. This left 12 of the 27 polymorphisms investigated originally: rs2372536 (*ATIC*), rs4673993 (*ATIC*), rs9344 (*CCND1*), rs1800909 (*GGH*), rs3758149 (*GGH*), rs2236225 (*MTHFD1*), rs1801133 (*MTHFR*), rs1801394 (*MTRR*), rs2306283 (*SLCO1B1*), rs4149056 (*SLCO1B1*), rs1142345 (*TPMT*), and rs1800460 (*TPMT*).

### Ethnic classification of Amerindian populations

The Amerindian ancestry of the individuals included in the present study was confirmed using the set of 61 Ancestry-Informative Markers (AIMs) described by Santos et al.^12^ and Ramos et al.^13^. The proportion of ancestry was estimated in STRUCTURE v2.3.3, assuming the contribution of three parental populations, African, European, and Native American. Amerindians were defined as having at least 95% native American ancestry.

All the individuals included in the present study had more than 95% native American ancestry. The overall mean genomic ancestry of the study population was 0.022 European, 0.014 African, and 0.964 native American (see supplementary Figure 3).

### Statistical analysis

The statistical analyses were run in the R v.3.4.0 program (R Foundation for Statistical Computing). The inter-population variability of the polymorphisms analyzed in the present study was evaluated using Wright’s Fixation Index (FST). The FST values were used to run a multidimensional scaling analysis to plot the genetic differentiation of the Amerindian population with those from the five continental populations being included for comparison. The differences between the genotype frequencies of the Amerindian populations and those from the 1,000 Genomes database were evaluated using Fisher’s exact test. The False Discovery Rate (FDR) was used to correct the multiple analyses^14^. A *p* ≤ 0.05 significance was considered for all analyses.

## Results

**Figure 1:**
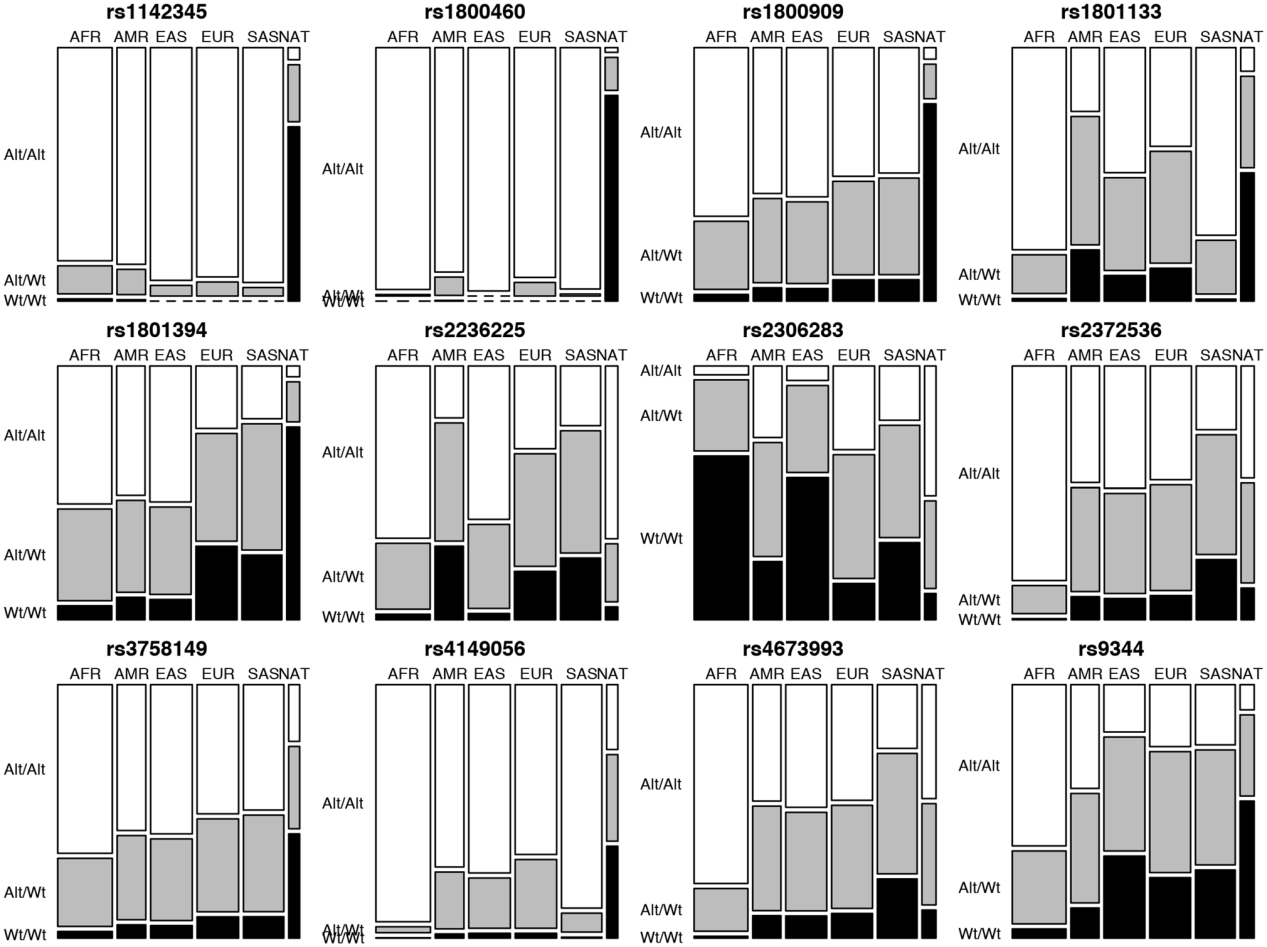
Mosaic plots of the distribution of the genotypes of the 12 markers investigated in the six study populations (AFR, AMR, EAS, EUR, SAS, and NAT). Genotypes: Alt/Alt = mutant homozygote, Alt/Wt = heterozygote, and Wt/Wt = wild homozygote.

**Figure 2:**
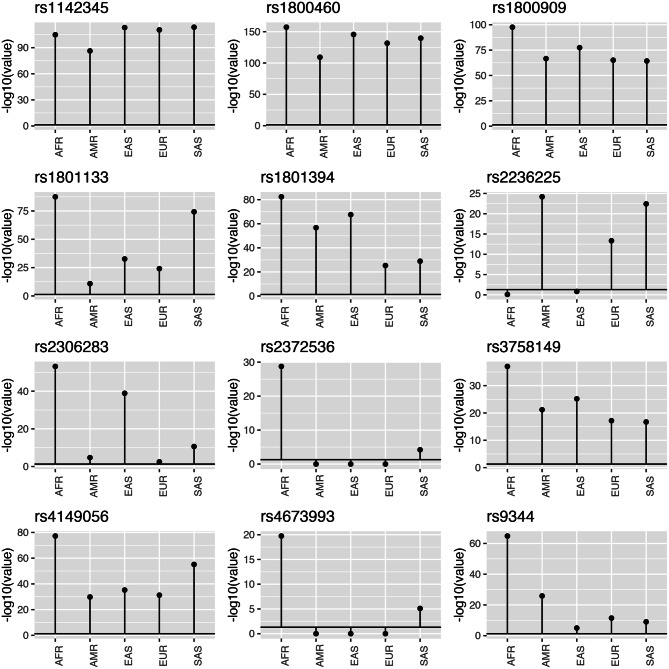
Plots of the adjusted *p* values (FDR) for the comparison of the 12 markers investigated in the present study between the NAT population and the five continental populations (AFR, AMR, EAS, EUR, SAS), based on Fisher’s exact test. The significance threshold (*p* = 0.05) is represented by the bold horizontal line.

The allele frequencies recorded for the 12 markers investigated in the NAT population and the continental populations (AFR, AMR, EAS, EUR, and SAS) are shown in Table 1, and the distribution of the genotypes is plotted in Fig. 1. Nine of the 12 markers investigated in the present study vary significantly in the NAT population in comparison with all five continental populations. These polymorphisms are rs9344_*CCND1*, rs1800909_*GGH*, rs3758149_*GGH*, rs1801133 _*MTHFR*, rs1801394_*MTRR*, rs2306283_*SLCO1B1*, rs4149056_*SLCO1B1*, rs1142345_*TPMT* and rs1800460_*TPMT* (Fig. 2, Table 2). The other three markers presented significant differences between the NAT population and only some of the continental populations. In the case of the rs2236225 polymorphism of the *MTHFD1* gene, for example, the NAT population was not significantly different (P > 0.05) from either the AFR or the EAS population, while rs23725336 (*ATIC* gene) was only significantly different from the AFR population, and rs4673993 (*ATIC*), only from the AFR and SAS populations.

**Table 1:** Allele frequencies of the markers investigated in the different study populations (AFR, AMR, EAS, EUR, SAS, and NAT).

Gene	SNP, ID	Reference allele	Frequency in population:					
			NAT	AFR	AMR	EAS	EUR	SAS
*TPMT*	rs1142345	T	0.873	0.933	0.942	0.978	0.971	0.982
*TPMT*	rs1800460	C	0.946	0.997	0.959	1	0.972	0.995
*MTHFR*	rs1801133	G	0.712	0.909	0.525	0.704	0.635	0.881
*MTRR*	rs1801394	A	0.903	0.754	0.719	0.737	0.477	0.475
*MTHFD1*	rs2236225	G	0.174	0.842	0.455	0.801	0.570	0.495
*GGH*	rs1800909	A	0.883	0.833	0.772	0.780	0.720	0.713
*GGH*	rs3758149	G	0.581	0.833	0.772	0.780	0.721	0.713
*ATIC*	rs4673993	T	0.323	0.905	0.693	0.706	0.686	0.509
*ATIC*	rs2372536	C	0.336	0.934	0.691	0.707	0.683	0.507
*SLCO1B1*	rs2306283	A	0.290	0.182	0.527	0.238	0.597	0.452
*SLCO1B1*	rs4149056	T	0.548	0.986	0.865	0.876	0.838	0.957
*CCND1*	rs9344	G	0.735	0.812	0.651	0.428	0.502	0.483

**Table 2:** Adjusted *p* values (FDR) for the comparison of the 12 markers investigated in the present study between the NAT population and the five continental populations (AFR, AMR, EAS, EUR, SAS), based on Fisher’s exact test.

Gene	SNP, ID	Reference allele	Adjusted *p* value (FDR) for the NAT population *versus*:				
			AFR	AMR	EAS	EUR	SAS
*TPMT*	rs1142345	T	1.48e−105	5.44e−87	8.46e−114	3.82e−111	3.07e−114
*TPMT*	rs1800460	C	6.47e−158	5.74e−110	2.64e−146	2.24e−132	2.24e−140
*MTHFR*	rs1801133	G	3.17e−88	1.58e−11	2.31e−33	8.71e−25	5.72e−75
*MTRR*	rs1801394	A	4.35e−83	2.04e−57	2.69e−68	4.89e−26	1.22e−29
*MTHFD1*	rs2236225	G	8.00e−01	6.73e−25	1.45e−01	4.58e−14	3.78e−23
*GGH*	rs1800909	A	2.69e−98	2.59e−67	3.86e−78	8.70e−66	5.40e−65
*GGH*	rs3758149	G	9.35e−38	6.70e−22	6.57e−26	6.77e−18	1.96e−17
*ATIC*	rs4673993	T	1.85e−20	1.00e+00	1.00e+00	1.00e+00	7.89e−06
*ATIC*	rs2372536	C	1.80e−29	1.00e+00	1.00e+00	1.00e+00	6.31e−05
*SLCO1B1*	rs2306283	A	7.09e−54	1.89e−05	1.33e−39	3.26e−03	2.37e−11
*SLCO1B1*	rs4149056	T	6.09e−78	1.48e−30	5.85e−36	5.73e−32	7.22e−56
*CCND1*	rs9344	G	1.49e−65	1.39e−26	1.04e−05	3.65e−12	1.02e−09

**Figure 3:**
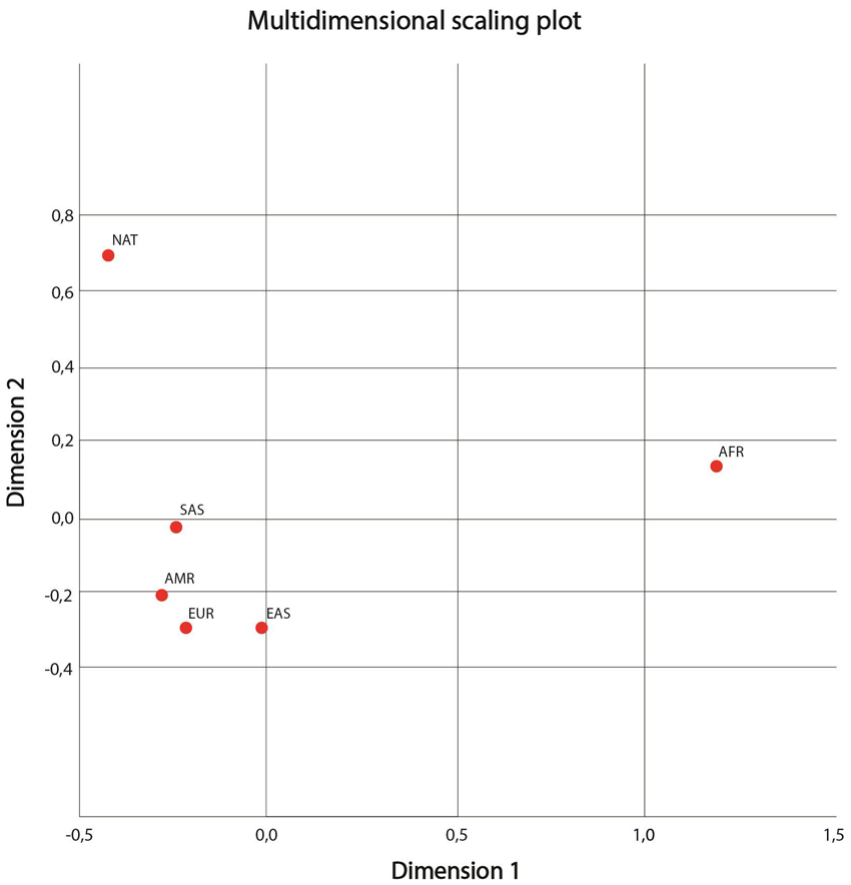
Plot of the results of the multidimensional spacing analysis of the genetic profiles of the six study populations investigated in the present study (AFR, AMR, EAS, EUR, SAS, and NAT), based on the 12 polymorphisms selected for analysis.

The plot of the Multidimensional Scaling (MDS) analysis of the FST values of the pairwise comparisons of the polymorphisms is presented in Fig. 3. The distribution of the points reveals that both the Amerindian (NAT) and the African (AFR) populations have distinct genetic profiles from those of the other populations (AMR, EAS, EUR, and SAS) included in the analysis, given their isolation in the extremities of the plot. The genetic profile of the Amerindian population is most similar to those of the American (AMR) and South Asian (SAS) populations.

**Figure 4:**
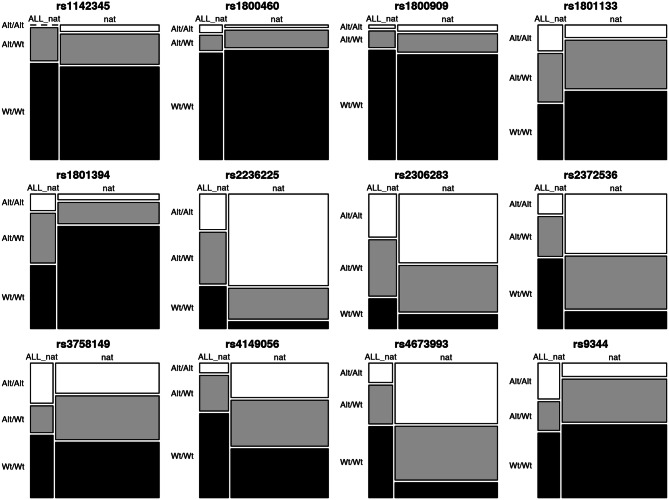
Mosaic plots of the genotype distribution of the 12 markers investigated in Amerindian populations (NAT) and ALL patients (ALL_NAT). Genotypes: Alt/Alt = mutant homozygote, Alt/Wt = heterozygote, and Wt/Wt = wild homozygote. The *p* values from right to left are: 1; 1; 1; 0.292; 0.07; ≤ 0.001; 0.154; ≤ 0.001; 0.986; 0.051; ≤ 0.001; 0.112.

An additional analysis was performed to show whether the allelic variations of the 12 markers investigated in traditional Amerindian populations would show the same genetic profile in ALL patients with a high Amerindian genetic contribution. Thus, the genotypic distribution of these markers was compared between the Amerindian populations investigated in this study (NAT) and leukemic patients (ALL_NAT) with an average of 50% of Amerindian genetic ancestry (see Supplementary Table 1). Differences between genotypic frequencies were observed using Fisher's exact test and FDR was used to correct multiple analyses. The results are shown in Fig. 4. Of the 12 markers, eight of them (rs1142345, rs1800460, rs1800909, rs1801133, rs2306283, rs3758149, rs4149056 and rs9344) did not show a statistically significant difference, which indicates that the two populations are genetically similar.

## Discussion

Disparities among ethnic groups in the treatment of ALL have been reported widely^5–10^, and the evidence indicates, in particular, that Hispanic children have the worst response to the therapeutic treatment of this cancer^5–7,9^. While this tendency has been associated partially with the contribution of native American ancestry in admixed Hispanic populations^9,15–17^, no previous study had focused specifically on the genetic markers associated with the treatment of ALL in Brazilian Amerindian populations. This reinforces the need for the investigation of the molecular variants in Amerindian populations, not only for a better understanding of the genetic variability of this population, but also to provide important empirical insights for the development of personalized protocols for the treatment of ALL in this ethnic group, as well as in other groups with a high level of Amerindian admixture. The findings of the present study should thus contribute to the improvement of the efficacy and security of the medication used to treat ALL in the Amerindian populations of the Amazon region.

Here, the distribution of the allele and genotype frequencies of 12 important pharmacogenomic markers related to 6-MP and MTX, the principal drugs used in the treatment of ALL, was compared between Amerindian (NAT) populations and five representative populations from the 1,000 Genomes database, using Fisher’s exact test with the standard statistical correction (FDR) being used to avoid misleading correlations.

The frequencies of all the pharmacogenomic markers analyzed in the present study were significantly different in the NAT population in comparison with all (in nine cases) or most of the continental populations (AFR, AMR, EAS, EUR, and SAS). The least differentiated marker was rs23725336 (*ATIC*), which varied significantly only between the NAT and AFR populations, while the frequency of rs4673993 (*ATIC*) of the NAT population was significantly different only from those of the AFR and SAS populations. The third marker, rs2236225 (*MTHFD1*), was significantly different in the AMR, EUR, and SAS populations.

The MDS analysis based on the FST values indicated that the Amerindian (NAT) and African (AFR) populations were the most genetically distinct. This analysis also found that the genetic profile of the Amerindian (NAT) population is most similar to those of the American (AMR) and South Asian (SAS) populations, which is consistent with their closer links through the history of human migrations^18,19^.

The 6-MP drug is an anti-metabolic purine analog that interferes with the biosynthesis of nucleic acids and is a key medication for the successful treatment of children with ALL. This drug is the only anti-leukemia medication used in the treatment of ALL for which the FDA (Food and Drug Administration) recommends the prior determination of the patient’s genetic profile prior to the administration of the drug, to allow for the adjustment of the treatment. A number of studies have shown an association between the rs1142345 and rs1800460 markers of the *TPMT* gene and an increased susceptibility to grave toxicity during the treatment of ALL with 6-MP^20–22^.

The MTX is a folate inhibitor that is an important component of virtually all the pediatric ALL treatment protocols. The association between the *MTHFR*, *MTHFD1*, and *MTRR* genes and the treatment of ALL with MTX has been the focus of a number of studies. In particular, the rs1801133 (*MTHFR*)^23,24^, rs2236225 (*MTHFD1*)^25,26^, and rs1801394 (MTRR)^27,28^ polymorphisms have all been associated with relapses and toxicity in MTX-based treatments.

In a Genome-Wide Association (GWA) study, Trevino et al.^29^ analyzed a number of polymorphisms of the *SLCO1B* gene, including rs4149056, and found a strong association with toxicity to MTX. Other studies have also recorded an association between the rs2306283 polymorphism of the *SLCO1B* gene and toxicity in MTX treatment^30–32^.

A number of other studies have found an association between variants of other genes involved the metabolic pathways of MTX and toxicity in treatments based on this drug. These variants include the rs2372536 and rs4673993 of the *ATIC* gene, which is involved in the synthesis of purines^33–35^ and polymorphisms of genes, such as *CCND1* (rs9344), involved in the regulation of the cell cycle and the enzymes targeted by MTX^36,37^. In addition, the rs1800909 and rs3758149 polymorphisms of the *GGH* gene, which catalyzes the degradation of the active polyglutamates of the natural folate and the MTX, have also been identified as predictors of toxicity in MTX^38,39^.

Our work aimed to provide information on the distribution of pharmacogenomic variants relevant to the treatment of ALL in traditional Amerindian populations. It would be interesting to demonstrate whether the results observed for the markers investigated here would show the same profile in leukemic patients of the same ethnic group. However, samples from indigenous patients with ALL are difficult to obtain, given the rarity of the disease and due to these populations living in remote rural areas that are difficult to access, which reflects a difficulty in care and clinical monitoring, coupled with the cultural factor, in which the treatment is, mostly, based on traditional indigenous medicine. To get around this issue, we used, for statistical comparison purposes, sample data from 42 patients with ALL, from the Brazilian Amazon metropolitan region, who had a high Amerindian genetic contribution. Of the 12 markers investigated, eight of them (rs1142345, rs1800460, rs1800909, rs1801133, rs2306283, rs3758149, rs4149056 and rs9344) did not show statistically significant differences between traditional Amerindian populations (NAT) and patients with ALL (ALL_NAT). This outcome may allow us to suggest a genetic similarity between the two groups. These markers were investigated for the risk of developing severe toxicity in a study previously carried out by our research group involving an admixed population of the Brazilian Amazon region^10^. In this study, the G allele of the rs2306283 variant of the *SLCO1B1* gene was associated with an almost three-fold increase in the risk of developing severe central nervous system toxicity during the consolidation phase of ALL treatment. In addition, a strong, but not significant, association was identified between the rs1801133 variant of *MTHFR* gene and the risk of developing severe toxicity in the disease consolidation phase.

In the current study, four markers were statistically different between Amerindian populations and samples of ALL patients (rs1801394, rs2236225, rs2372535 and rs4673993). The statistical difference observed for these markers could be due to their strong relationship with the susceptibility to ALL development. These polymorphisms were investigated in a study developed by our research group^40^ involving miscegenated populations from the Amazon. The rs2236225 variant of *MTHFD1* gene was related to a protective effect against the development of ALL, while the rs4673993 variant of *ATIC* gene was associated with a risk effect of developing the disease. In a meta-analysis developed by Fang et al.^41^, the GG genotype of the rs1801394 variant of *MTRR* gene was associated with a reduced risk of developing ALL in a Caucasian population.

The results of the present study indicate that the genotypic distribution of the markers analysed here in the Amerindian population is distinct from that of the other populations included here for comparison, from Africa, Europe, the Americas, and southern and eastern Asia. These findings highlight the unique nature of the genetic profile of the indigenous population of the Brazilian Amazon region, which also implies a distinct profile of efficacy and toxicity in the treatment of ALL in these populations, as well as other populations in Brazil formed through a high degree of admixture with this indigenous group, such as the Brazilian Amazonian populations.

## Supplementary information

Supplementary information
