# Supplementary material for: Characterization of pharmacogenetic markers related to Acute Lymphoblastic Leukemia toxicity in Amazonian native Americans population
Source: Sci Rep. 2020 Jun 24;10:10292. doi: 10.1038/s41598-020-67312-y (PMC7314857; doi:10.1038/s41598-020-67312-y)
Supplement: Supplementary file 1 — Supplementary information [file 41598_2020_67312_MOESM1_ESM.docx]

Characterization of pharmacogenetic markers related to Acute Lymphoblastic Leukemia toxicity in Amazonian Native Americans population.

Darlen Cardoso de Carvalho^1^, Alayde Vieira Wanderley^1,3^, André Mauricio Ribeiro dos Santos^2^, Fabiano Cordeiro Moreira^1^, Roberta Borges Andrade de Sá^1^, Marianne Rodrigues Fernandes^1^, Antonio André Conde Modesto^1^, Tatiane Piedade de Souza^1^, Amanda de Nazaré Cohen Lima de Castro^1^, Luciana Pereira Colares Leitão^1^, Juliana Carla Gomes Rodrigues^1^, Artur Luiz da Costa da Silva^4^, Joao Farias Guerreiro^2^, Sidney Santos^1,2^, André Salim Khayat^1^, Paulo Pimentel de Assumpção^1,5^, Ney Pereira Carneiro dos Santos ^1,2*^

^1^ Oncology Research Nucleus, Universidade Federal do Pará, Belém, Pará, PA, Brazil.

^2^ Human and Medical Genetics Laboratory, Instituto de Ciências Biológicas, Universidade Federal do Pará, Belém, Pará, PA, Brazil.

^3^ Ophir Loyola Hospital, Departamento de Pediatria, Belém, Pará, PA, Brazil.

^4^ Genomics and Bioinformatics Laboratory, Instituto de Ciências Biológicas, Universidade Federal do Pará, Belém, Brazil.

^5^ João de Barros Barreto University Hospital, Universidade Federal do Pará, Belém, PA, Brazil.

*Correspondence: Ney Pereira Carneiro dos Santos

Email: npcsantos.ufpa@gmail.com

Hospital Universitário João de Barros Barreto - Núcleo de Pesquisa em Oncologia, 2º piso da Unidade de Alta Complexidade em Oncologia. Av. Mundurucus, 4487, Guamá, 66073-005, Belém –PA, Brazil. Tel: +55 (91) 3201-6778.

**
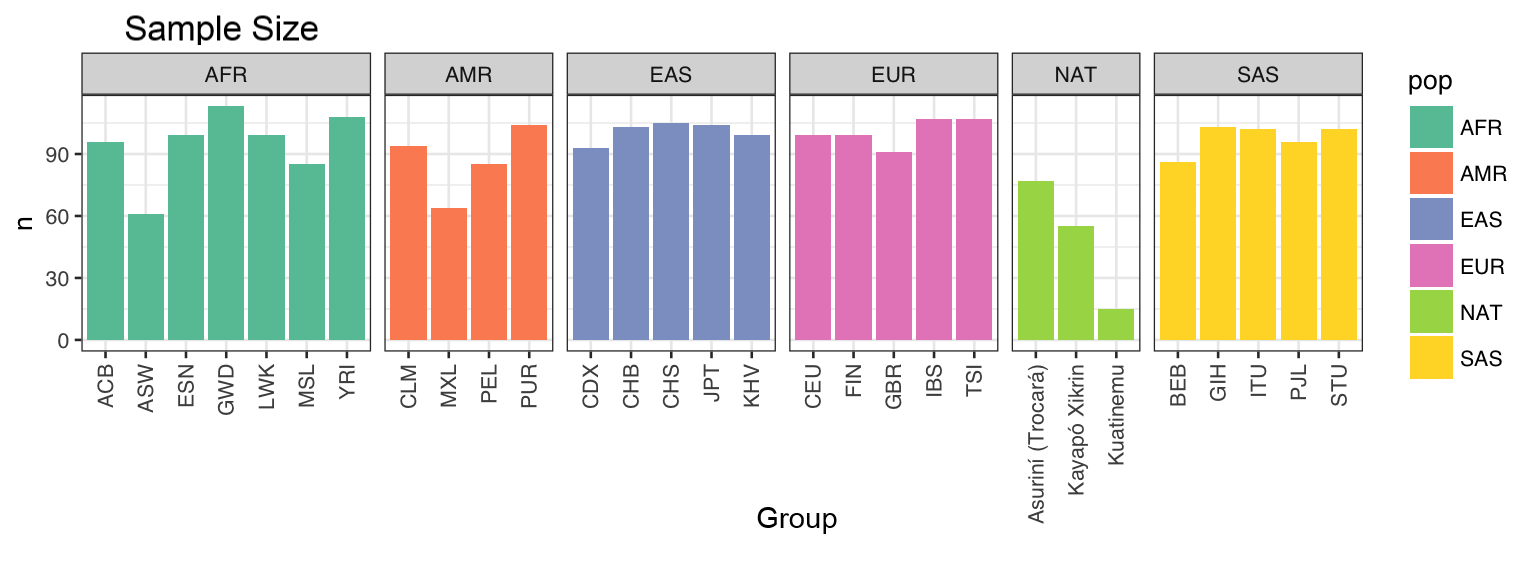
**

**Supplementary Figure 1**. Sample sizes. Seven populations from the African (AFR) region were included in the sample from this continent (ACB, ASW, ESN, GWD, LWK, MSL, and YRI), while five populations (CEU, FIN, GBR, IBS and TSI) represented Europe (EUR). Asia was divided into two groups, East Asia (EAS), with five populations (CDX, CHB, CHS, JPT and KHV), and South Asia (SAS), also with five populations (BEB, GIH, ITU, PJL and STU). The Americas (AMR) were represented by four populations (CLM, MXL, PEL and PUR). The Amerindians of the Amazon region were represented by three populations (Asurini do Koatinemo, Asurini do Trocará, and Kayapó-Xicrin).


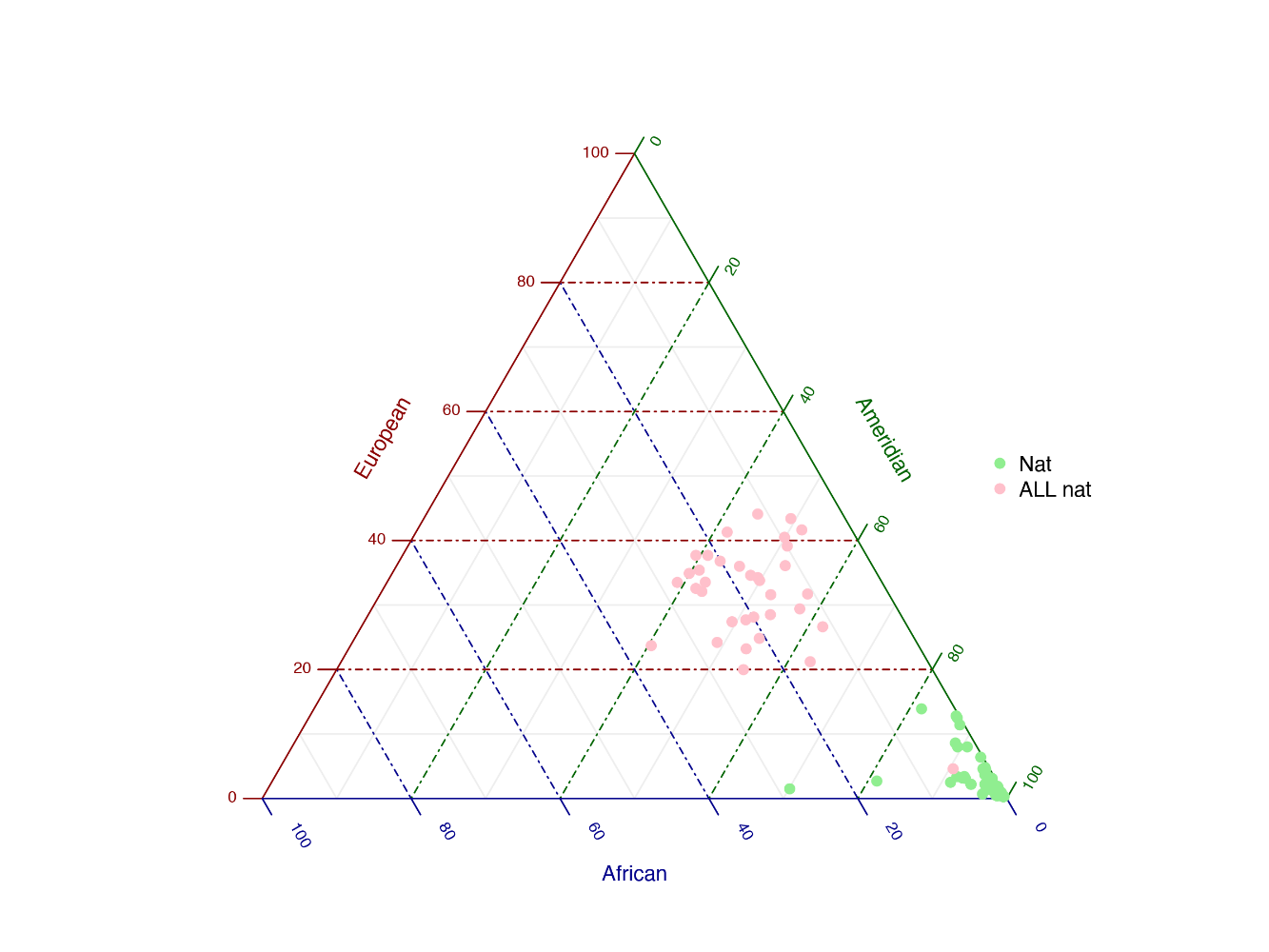


**Supplementary Figure 2**. Triangular graph of ancestry analysis of ALL patients (ALL_NAT), represented by pink dots, and Amerindian populations (NAT), represented by green dots. The estimates were obtained by the STRUCTURE program, based on 61 ancestry informative markers.


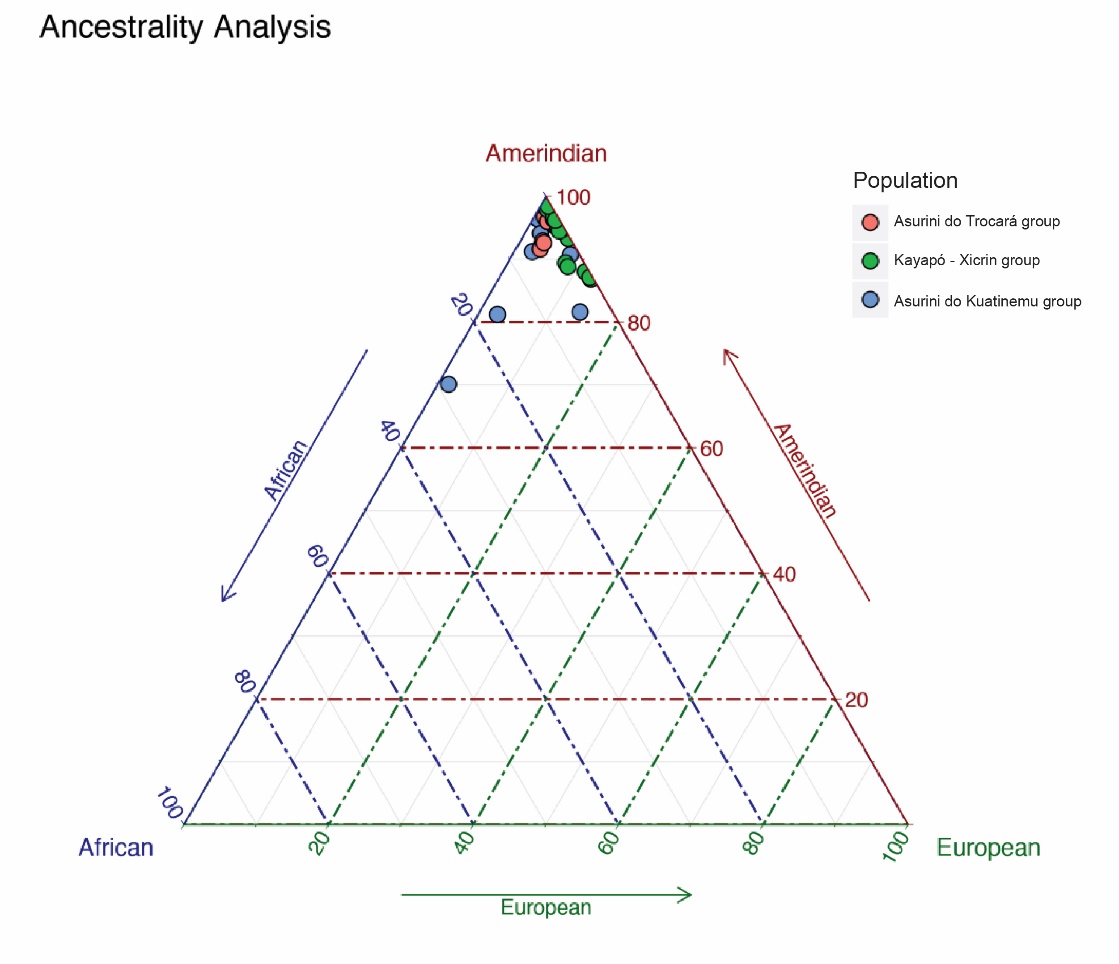


**Supplementary Figure 3**. Triangular plot of the ancestry analysis of the Amerindian (NAT) populations analyzed in the present study. The estimates were obtained in the STRUCTURE program, based on 61 ancestry-informative markers. The red dots represent the individuals of the Asurini do Trocará group, the green dots, those of the Kayapó-Xicrin group, and the blue dots, those of the Asurini do Koatinemo group.

**Supplementary Table 1**. Characteristics of the patients in the present study.

| **Variable** | **n (%)** |
| --- | --- |
| **Number of patients** | 42 |
| **Sex** |  |
| Male | 25 (59.5) |
| Female | 17 (40.5) |
| **Mean age at diagnosis (years, SD±**^1^) | 5.02±3.25 |
| **Leukometry at diagnosis (µL)** |  |
| <50,000 | 32 (76.2) |
| ≥50,000 | 10 (23.8) |
| **Risk group** |  |
| Low | 19 (45.2) |
| Medium | 4 (9.6) |
| High | 19 (45.2) |
| **Mean genetic ancestry (SD±**^1^) |  |
| European | 0.32±0.08 |
| African | 0.18±0.07 |
| Amerindian | 0.50±0.09 |
| **Chromosomal translocations** |  |
| Absent | 28 (66.7) |
| *BCR-ABL* | 3 (7.1) |
| *ETV6-RUNX1* | 3 (7.1) |
| *TCF3-PBX1* | 7 (16.7) |
| *SIL-TAL* | 1 (2.7) |
| **Toxicity (3rd–4th degree) in the consolidation phase of the treatment** |  |
| Any | 28 (66.7) |
| Gastrointestinal | 11 (26.2) |
| Hematological | 3 (7.1) |
| Central Nervous System | 8 (19) |
| Infectious | 19 (45.2) |
| **Toxicity (3rd–4th degree) in the maintenance phase of the treatment** |  |
| Any | 23 (54.8) |
| Gastrointestinal | 9 (19) |
| Hematological | 15 (35.7) |
| Central Nervous System | 2 (4.8) |
| Infectious | 4 (9.5) |

^1^SD = Standard Deviation.

**Supplementary Table 2**. Characteristics of the molecular markers selected for analysis in the present study.

| **Gene** | **SNP, ID** | **Associated drug** | **Allele** | **Function** | **Amino acid shift** | **Chr** |
| --- | --- | --- | --- | --- | --- | --- |
| *ABCC1* | rs28364006 | MTX | A > G | Missense | Thr1337Ala | 16 |
| *ABCC2* | rs717620 | MTX | C > T | 5' UTR | - | 10 |
| *ABCC3* | rs9895420 | MTX | T > A | 5' Flanking | - | 17 |
| *AMPD1* | rs17602729 | MTX | G > A | Stop Codon | Gln45Ter | 1 |
| *ATIC* | rs2372536 | MTX | C > G | Missense | Thr116Ser | 2 |
| *ATIC* | rs4673993 | MTX | T > C | Splicing region | - | 2 |
| *CCND1* | rs9344 | MTX | G > A | Synonymous | Pro241Pro | 11 |
| *GGH* | rs11545078 | MTX | G > A | Missense | Thr151Ile | 8 |
| *GGH* | rs1800909 | MTX | A > G | Missense | Cys6Arg | 8 |
| *GGH* | rs3758149 | MTX | G > A | 5' Flanking | - | 8 |
| *ITPA* | rs1127354 | MTX | C > A | Missense | Pro15Thr | 20 |
| *MTHFD1* | rs2236225 | MTX | G > A | Missense | Arg653Gln | 14 |
| *MTHFR* | rs1801133 | MTX | G > A | Missense | Ala222Val | 1 |
| *MTRR* | rs1801394 | MTX | A > G | Missense | Ile22Met | 5 |
| *NALCN* | rs7992226 | MTX | A > G | Intronic | - | 13 |
| *NOS3* | rs1799983 | MTX | T > G | Missense | Asp298Glu | 7 |
| *SHMT1* | rs1979277 | MTX | G > A | Missense | Leu435Phe | 17 |
| *SLCO1B1* | rs2306283 | MTX | A > G | Missense | Asn130Asp | 12 |
| *SLCO1B1* | rs4149015 | MTX | G > A | 5' Flanking | - | 12 |
| *SLCO1B1* | rs4149056 | MTX | T > C | Missense | Val174Ala | 12 |
| *TLR4* | rs4986790 | MTX | A > G | Missense | Asp99Gly | 9 |
| *TNFAIP3* | rs6920220 | MTX | G > A | Intergenic | - | 6 |
| *TPMT* | rs1800460 | 6-MP | C > T | Missense | Ala154Thr | 6 |
| *TPMT* | rs1800462 | 6-MP | C > G | Missense | Ala80Pro | 6 |
| *TPMT* | rs1142345 | 6-MP | T > C | Missense | Tyr240Cys | 6 |
| *TPMT* | rs12201199 | 6-MP | A > T | Intronic | - | 6 |
| *TPMT* | rs56161402 | 6-MP | C > T | Missense | Arg215His | 6 |
